# Social determinants of health and lifestyle in the progression of degenerative bone and joint diseases: a prospective multi-state analysis

**DOI:** 10.1186/s12889-026-28916-7

**Published:** 2026-08-07

**Authors:** Xiaofeng Ma, Xin Song, Yanqiu Zou, Zilan Chen, Yan Su, Bowen Lei, Di Zhang, Bin Yang, Yang Qu, Tao Han, Maoyao Xia, Yangdan Zhong, Mengyu Fan, Xia Jiang, Ben Zhang

**Affiliations:** 1 Department of Epidemiology and Biostatistics, West China School of Public Health and West China Fourth Hospital, Sichuan University, Chengdu, Sichuan China; 2 Department of Nutrition and Food Hygiene, West China School of Public Health, West China Fourth Hospital, Sichuan University, Chengdu, China; 3 Department of Clinical Neuroscience, Karolinska Institute, Stockholm, Sweden

**Keywords:** Degenerative bone and joint diseases, Multimorbidity, Social determinants of health, Lifestyle, Multi-state model, Cohort study

## Abstract

**Aims:**

The role of social determinants of health (SDOH) in the progression of degenerative bone and joint diseases (DBJDs) - from initial onset to multimorbidity and mortality - remains unclear. We aimed to assess the associations of SDOH with DBJDs progression, examine the mediating role of lifestyle, and explore their joint effects.

**Methods:**

A total of 283,146 participants from the UK Biobank were included. SDOH was constructed using 17 items including economic, education, healthcare, neighbourhood, and social domains, and categorized into favourable, intermediate, and unfavourable groups. The outcomes of interest were first DBJD (FDBJD), DBJM (the coexistence of at least two DBJDs), and all-cause mortality. Multi-state models were employed to estimate risks across five disease transitions.

**Results:**

During a median follow-up of 13.7 years, 43,172 participants developed FDBJD, of whom, 4,170 subsequently developed DBJM, and 18,831 died. Compared with favourable SDOH, unfavourable SDOH was associated with increased risks of all transitions, including from baseline to FDBJD (HR = 1.21, 95%CI = 1.18–1.24), from baseline to death (1.58 [1.52–1.65]), from FDBJD to DBJM (1.33 [1.23–1.44]), from FDBJD to death (1.64 [1.50–1.80]), and from DBJM to death (1.48 [1.17–1.87]). Economic stability and social and community context emerged as the most robust predictors. Lifestyle factors partially mediated the associations, with proportions of mediation ranging from 2.47% to 10.77%. Participants with unfavourable SDOH and lifestyle exhibited the highest risks, with significant additive interactions observed in transitions of death from baseline or after FDBJD.

**Conclusions:**

Unfavourable SDOH are strongly associated with progression and mortality of DBJDs, with effects that extend beyond individual lifestyle factors. These findings underscore the importance of addressing upstream social determinants, alongside lifestyle promotion, to reduce the burden of DBJDs and social inequalities in musculoskeletal health.

**Supplementary Information:**

The online version contains supplementary material available at https://doi.org/10.1186/s12889-026-28916-7.

## Introduction

Degenerative bone and joint diseases (DBJDs), primarily including osteoarthritis, osteoporosis, and intervertebral disc degeneration (IVDD), constitute a significant and growing public health burden worldwide [1]. These disorders are characterized by articular cartilage degradation, progressive bone loss, and chronic inflammation of periarticular soft tissues [2], typically leading to impaired joint function and ultimately causing pain and physical disability [3–5]. In ageing populations, the clinical burden of DBJDs is increasingly driven not only by disease onset but also by subsequent progression, characterized by the accumulation of multiple degenerative conditions and an elevated risk of premature mortality [1, 3, 6]. Identifying determinants of these disease trajectories is therefore central to improving long-term outcomes in bone health practice.

Beyond biological susceptibility, social determinants of health (SDOH), including economic stability, education access and quality, healthcare access and quality, neighbourhood and built environment, and social and community context [7, 8], are fundamental drivers of health inequalities and have been repeatedly linked to musculoskeletal outcomes. Individuals exposed to disadvantaged social conditions experience a higher prevalence of osteoarthritis, more severe pain and functional impairment, and poorer survival [9–11]. Socioeconomic gradients have also been observed in musculoskeletal care pathways, including access to specialist services and joint replacement surgery, highlighting the influence of structural determinants beyond clinical severity alone [12, 13]. Importantly, these disparities suggest that a substantial proportion of adverse musculoskeletal outcomes may be preventable if social disadvantage could be mitigated. However, most existing studies have been limited by focusing on isolated social risk factors—such as socioeconomic status or race/ethnicity—and by examining single DBJD conditions or discrete transitions, such as from disease onset to diagnosis or from DBJDs to mortality [11–16]. This fragmented approach leaves essential gaps in understanding how integrated SDOH dynamically influence progression of DBJDs from initial occurrence through multimorbidity and ultimately to death.

Lifestyle factors represent key modifiable exposures that may contribute to mitigate socially patterned inequalities in musculoskeletal health. Unhealthy behaviors are more common among socially disadvantaged groups and have been linked to increased risks of DBJDs and adverse prognosis [3–5], yet it remains unclear to what extent improving lifestyle could offset the excess burden attributable to adverse SDOH. Estimating the proportion of disease progression and mortality that could theoretically be avoided through improvements in social conditions and health behaviors is critical for informing population-level prevention strategies.

By utilizing a large population-based cohort, this study aimed to comprehensively evaluate the associations between SDOH and the progression of DBJDs from baseline health to first DBJD, subsequent multimorbidity, and ultimately to death. Furthermore, we examined the mediating role of lifestyle and quantified the potentially preventable burden associated with SDOH across stages of disease progression.

## Methods

### Study population

The UK Biobank (UKB) is a large prospective cohort study that recruited over 0.5 million participants aged 37–73 years from 22 assessment centers across the UK between 2006 and 2010. The participants were asked to complete touchscreen questionnaires, undergo physical measurements, and provide biological samples. Details have been described previously [17]. UKB had obtained ethics approval from the North West Multi-Centre Research Ethics Committee (approval number: 11/NW/0382) and had obtained informed consent from all participants. The present study was conducted under UKB application number 99,713.

**Fig. 1:**
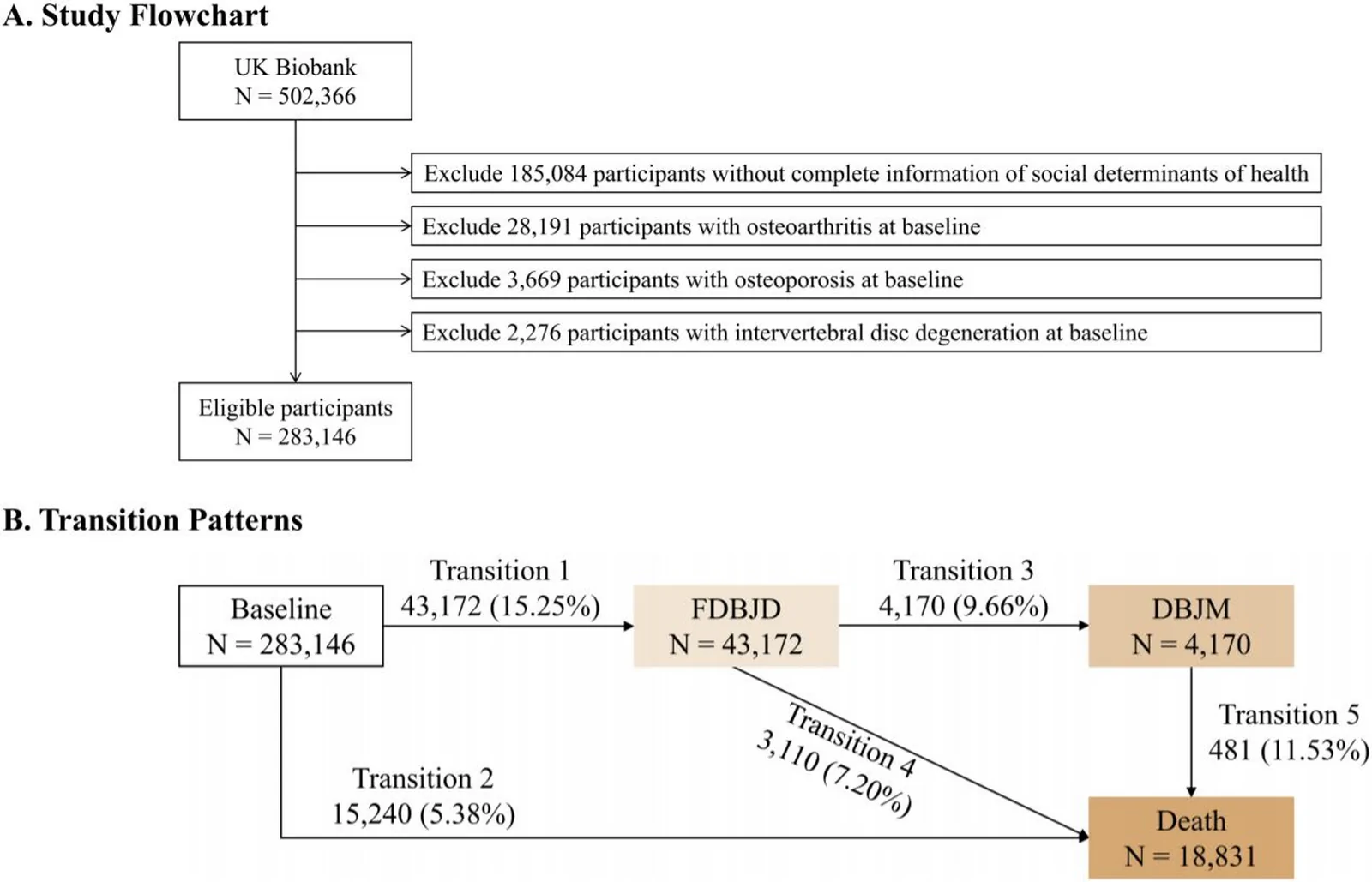
The flowchart (**A**) of participants selection and transition patterns (**B**) from baseline to FDBJD, then to DBJM, and ultimately to death. Notes: FDBJD, first degenerative bone and joint diseases; DBJM, degenerative bone and joint multimorbidity

In this study, we excluded participants with missing information on SDOH (*n* = 185,084), and those with osteoarthritis, osteoporosis, or IVDD at baseline (*n* = 34,136). Finally, 283,146 participants were included in the primary analysis (Fig. 1). For analyses involving lifestyle factors, we further restricted the sample to 229,595 participants with complete lifestyle data.

### Assessment of SDOH

In accordance with Healthy People 2030 objectives [7] and previous studies [18], SDOH were grouped into five domains: (i) economic stability (household income, employment status and area-level income deprivation), (ii) education access and quality (education attainment and area-level education deprivation), (iii) healthcare access and quality (area-level healthcare deprivation), (iv) neighbourhood and built environment (accommodation stability, area-level housing deprivation, crime score, and natural environment), and (v) social and community context (psychosocial problems, race or ethnicity, living alone or with partners, social support, social activity, social isolation, and emotional distress). Each SDOH component was dichotomized as advantaged (scored 0) or disadvantaged level (scored 1) using conventional cut points established in prior literature [18]. For individual SDOH domains, the component scores within each domain were summed, and participants were categorized into relatively favourable and unfavourable groups according to the median domain score. The overall SDOH score ranged from 0 to 17, with higher scores indicating less favourable SDOH. Participants were categorized into tertiles: favourable (0–5), intermediate (6–8), and unfavourable SDOH (9–17). Detailed definitions are provided in Table S1. As a sensitivity analysis, we also constructed weighted SDOH scores to account for potential differences in the contribution of individual SDOH components, with weights derived from *β* coefficients obtained from Cox proportional hazards models for all-cause mortality (Table S2). In UKB, weighted SDOH score = (*β*_1_ × SDOH_1_ + … + *β*_17_ × SDOH_17_) × (17/sum of *β* coefficients).

### Assessment of lifestyle factors

Based on established guidelines and previous studies [3–5], six lifestyle factors were used to calculate healthy lifestyle scores: smoking status, alcohol consumption, dietary pattern, physical activity, sleep duration, and BMI. Each lifestyle factor was scored as healthy (1 point) or unhealthy (0 point). A composite healthy lifestyle score was the sum of six individual scores, ranging from 0 to 6, with higher scores indicating a healthier lifestyle. Participants were classified into three groups: favourable (5–6), intermediate (3–4), and unfavourable lifestyle (0–2). Detailed definitions are shown in Table S3.

### Follow-up and ascertainment of outcomes

The outcomes of interest were incident FDBJD, DBJM, and all-cause mortality. FDBJD was defined as the first occurrence of any DBJDs (osteoarthritis, osteoporosis, or IVDD) during follow-up, and DBJM was defined as the subsequent coexistence of two or more of these conditions. Outcomes were identified through linkage to hospital admission data and death registry records, using International Classification of Diseases, Ninth and Tenth Revision (ICD-9 and ICD-10) codes. Detailed coding information [19, 20] is provided in Table S4. Follow-up time was calculated from the date of baseline to the diagnosis of outcomes, death, loss to follow-up, or end of follow-up (October 31, 2022), whichever came first.

### Assessment of covariates

Covariates included age, sex, smoking status, alcohol consumption, dietary pattern, physical activity, sleep duration, BMI, history of hypertension, diabetes, and hyperlipidemia. Detailed information is displayed in Table S5. In mediation and joint analyses involving lifestyle factors, lifestyle variables were not included as adjustment covariates.

### Statistical analysis

Descriptive characteristics were presented as means ± standard deviations (SD) for continuous variables and numbers (percentages) for categorical variables. Group differences were tested by one-way ANOVA for continuous variables and χ^2^ test for categorical variables. Missing data was imputed as median values for continuous variables and coded as independent categories for categorical variables.

Multi-state models [21, 22] were applied to estimate hazard ratios (HRs) and corresponding 95% confidence intervals (CIs) for associations between SDOH and transitions along the DBJDs trajectory. The overall SDOH score was analysed as both categorically and continuously, with HRs estimated per one-point increase. Five transitions were constructed: (i) from baseline to FDBJD; (ii) from baseline to death; (iii) from FDBJD to DBJM; (iv) from FDBJD to death; and (v) from DBJM to death (Fig. 1). For participants entering different stages on the same date, the entry date of the theoretically prior state was calculated as the date of the latter state minus 0.5 day. Furthermore, population-attributable fractions (PAFs) were calculated to estimate the proportion of transition events that could theoretically be avoided if all participants were at the reference exposure level. The reference categories were defined as follows: favourable SDOH for the overall SDOH analysis; the relatively favourable group for the analysis of each individual SDOH domain; and the group with both favourable SDOH and favourable lifestyle for the joint analysis.

Mediation analysis was performed to quantify the extent to which lifestyle potentially mediated associations between SDOH and DBJDs trajectory. This analysis employed a regression-based approach for survival data that decomposed the total effect into direct effect (pathways independent of lifestyle) and indirect effect (pathways through lifestyle), enabling calculation of the proportion mediated [23]. In the primary mediation analysis, SDOH and lifestyle were modeled as standardized continuous variables. As a sensitivity analysis, lifestyle was additionally modeled as an ordinal mediator based on the predefined lifestyle categories.

Joint associations of SDOH and lifestyle were evaluated, and additive interactions were assessed by calculating the relative excess risk due to interaction (RERI), attributable proportion (AP), and synergy index (S) with their 95% CIs. The lowest-risk stratum (favorable SDOH and favorable lifestyle) served as the reference category [24].

Sensitivity analyses were conducted to evaluate the robustness of our results: (i) excluding participants with missing covariates; (ii) excluding events occurring within the first 6 months and first year of follow-up; (iii) excluding participants who entered different states on the same date; (iv) calculating the entry date of the prior state using different time intervals (1, 30, and 365 days) for participants who entered different states on the same day; (v) a weighted SDOH score was constructed to test the robustness of the results; (vi) to mitigate potential information loss from dichotomizing SDOH, we reclassified SDOH according to the following approach: SDOH derived from questionnaires were categorized into three or more groups based on the original response options, while those obtained from area-level scores were classified into three categories according to the tertiles of their distributions (Table S6); (vii) excluding “psychosocial problems” and “emotional distress” from the SDOH score; (viii) performing multiple imputation for SDOH and covariates with missing data.

All statistical analyses were conducted using R software (version 4.4.2), and two-sided *P* < 0.05 considered statistically significant.

## Results

### Descriptive results

As shown in Table 1, this study included 283,146 participants with a mean age of 54.9 years (SD = 8.0), of whom 52.2% were female. Participants in the unfavourable SDOH group were more likely to be younger and women, with higher prevalence of hypertension, diabetes, and hyperlipidemia. They also tended to be current smokers, have poorer dietary quality, lower levels of physical activity, suboptimal sleep duration, and higher BMI.

**Table 1:** Baseline characteristics of participants in the UK Biobank by social determinants of health

Baseline characteristics	Total	Social determinants of health		
		Favourable [0–5]	Intermediate [6–8]	Unfavourable [9–17]
No. of participants	283,146	107,773	104,024	71,349
Age, years	54.9 ± 8.0	56.3 ± 7.8	54.7 ± 8.0	53.0 ± 7.9
Female, n (%)	147,899 (52.2)	53,448 (49.6)	55,320 (53.2)	39,131 (54.8)
Smoking status, n (%)				
Never	160,876 (56.8)	66,010 (61.2)	58,986 (56.7)	35,880 (50.3)
Previous	94,672 (33.4)	35,620 (33.1)	35,691 (34.3)	23,361 (32.7)
Current	27,177 (9.6)	6,012 (5.6)	9,194 (8.8)	11,971 (16.8)
Heavy alcohol drinking, n (%)	135,420 (47.8)	60,275 (55.9)	49,594 (47.7)	25,551 (35.8)
Unhealthy diet, n (%)	144,903 (51.2)	52,073 (48.3)	53,132 (51.1)	39,698 (55.6)
Low physical activity, n (%)	44,130 (15.6)	15,278 (14.2)	16,580 (15.9)	12,272 (17.2)
Sleep duration ≤ 6 or ≥ 9 h/d, n (%)	67,750 (23.9)	20,941 (19.4)	24,578 (23.6)	22,231 (31.2)
BMI	27.1 ± 4.6	26.5 ± 4.1	27.1 ± 4.6	27.8 ± 5.3
History of hypertension, n (%)	69,045 (24.4)	24,551 (22.8)	24,958 (24.0)	19,536 (27.4)
History of diabetes, n (%)	12,048 (4.3)	3,485 (3.2)	4,258 (4.1)	4,305 (6.0)
History of Hyperlipidemia, n (%)	31,159 (11.0)	11,309 (10.5)	11,392 (11.0)	8,458 (11.9)

Data are presented as mean ± standard deviation or n (%). For dichotomous variables, only the indicated category is presented

*Abbreviations*: *BMI* Body mass index, *h/d* Hours/day, *SDOH* Social determinants of health

All *P* values for comparisons across the SDOH groups were < 0.001

During a median follow-up period of 13.7 years (interquartile range: 13.0-14.4 years), 43,172 (15.25%) participants developed FDBJD, while 15,240 (5.38%) died without a DBJDs diagnosis. Among all incident FDBJD cases, 3,110 (7.20%) died without experiencing DBJM, and 4,170 (9.66%) developed DBJM, and afterward, 481 (11.53%) died from all causes (Fig. 1). Participants with unfavourable SDOH tended to have increased risks of developing FDBJD, DBJM, and death (Table S7-S9).

### Multi-state associations between SDOH and transitions in the DBJDs trajectory

**Fig. 2:**
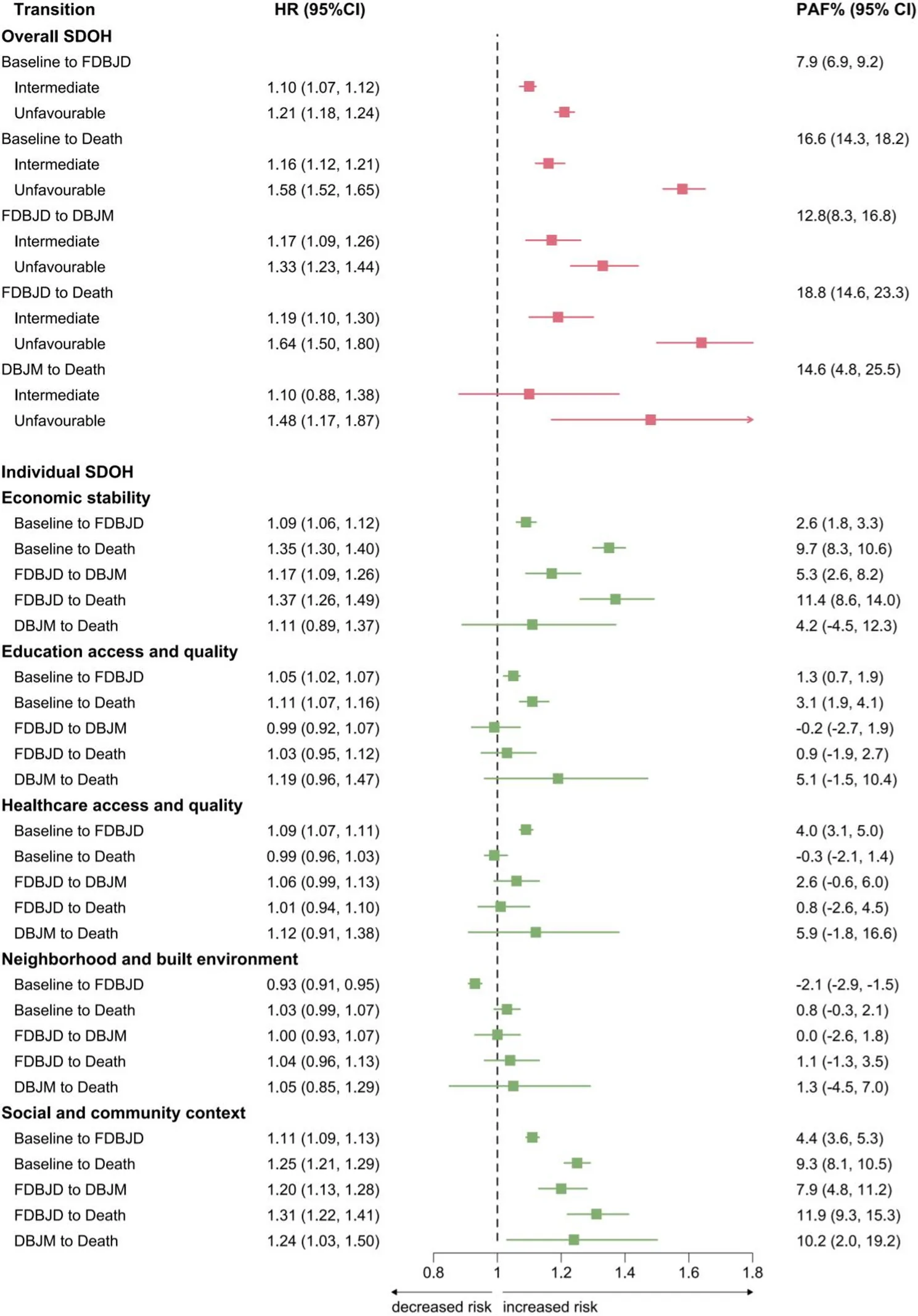
Associations of overall and individual social determinants of health with different transitions by multi-state model. Models were adjusted for age, sex, smoking status, alcohol drinking, healthy diet patterns, physical activity, sleep duration, BMI, history of hypertension, diabetes, and hyperlipidemia. Overall SDOH was categorized into favourable, intermediate, and unfavourable groups. For each individual SDOH domain, participants were categorized into relatively favourable and unfavourable groups according to the median domain score, with the favourable group serving as the reference. Notes: SDOH, social determinants of health; FDBJD, first degenerative bone and joint diseases; DBJM, degenerative bone and joint multimorbidity; BMI, body mass index; HR, hazard ratio; CI, confidence interval; PAF, population attributable fraction

Figure 2 presents the associations of overall and individual SDOH with transitions along the DBJDs trajectory, together with corresponding PAFs. Compared with participants with favourable SDOH, participants with unfavourable SDOH had consistently higher risks across all transitions. Specifically, unfavourable SDOH was associated with a 21% increased risk of developing FDBJD from baseline (HR = 1.21, 95%CI = 1.18–1.24) and a 33% increased risk of progressing from FDBJD to DBJM (HR = 1.33, 95%CI = 1.23–1.44). Stronger associations were observed for mortality transitions, with HRs of 1.58 (95%CI = 1.52–1.65) for death from baseline, 1.64 (95%CI = 1.50–1.80) for death after FDBJD, and 1.48 (95%CI = 1.17–1.87) for death following DBJM. Similar patterns were observed when SDOH was analysed as a continuous score (Table S10). PAF analysis showed that, under the hypothetical favourable-SDOH reference scenario, the estimated PAFs were 7.9% (95%CI = 6.9%-9.2%) for incident FDBJD, 16.6% (95%CI = 14.3%-18.2%) for death from baseline, 12.8% (95%CI = 8.3%-16.8%) for progression from FDBJD to DBJM, 18.8% (95%CI = 14.6%-23.3%) for death after FDBJD, and 14.6% (95%CI = 4.8%-25.5%) for death following DBJM.

Among individual SDOH components, economic stability and social and community context showed the strongest and most consistent associations across transitions (Fig. 2), particularly for mortality outcomes, with HRs ranging from 1.09 to 1.37 and 1.11 to 1.31, respectively. In contrast, education access and quality (HR = 1.05, 95%CI = 1.02–1.07) and healthcare access and quality (HR = 1.09, 95%CI = 1.07–1.11) were primarily associated with incident FDBJD and, to a lesser extent, baseline mortality. No significant association was observed for neighborhood or built environment across disease transitions.

### Mediation analyses of lifestyle in associations between SDOH and DBJDs trajectory

Mediation analyses revealed that lifestyle factors partially mediated the associations between SDOH and the DBJDs trajectory, although the magnitude of mediation varied by stage (Table 2). The largest proportion mediated was observed for incident FDBJD (10.77%, 95%CI = 9.55%-12.14%). For mortality transitions, lifestyle mediated 6.93% (95%CI = 6.13%-7.84%) of the association with death from baseline, 5.37% (95%CI = 3.35%-7.60%) with death after FDBJD, and 9.00% (95%CI = 2.15%-23.05%) with death following DBJM. The mediation for progression from FDBJD to DBJM was minimal and not statistically significant. Similar results were observed when lifestyle was modeled as an ordinal mediator (Table S11).

**Table 2:** The mediating role of lifestyle in the association of SDOH with transitions in the DBJDs trajectory

Transitions	Proportion Mediated, (95% CI)
Baseline to FDBJD	10.77% (9.55%, 12.14%)
Baseline to Death	6.93% (6.13%, 7.84%)
FDBJD to DBJM	2.47% (-3.18%, 5.37%)
FDBJD to Death	5.37% (3.35%, 7.60%)
DBJM to Death	9.00% (2.15%, 23.05%)

Models were adjusted for age, sex, history of hypertension, diabetes, and hyperlipidemia. The exposure variable was SDOH, the mediator was lifestyle, and the outcome variable was occurrence of the transition event. SDOH and lifestyle were analyzed as standardized continuous variables, expressed per 1-SD increase. The model from the exposure variable to the mediator variable was implemented using a linear regression model, while the model from the mediator variable to the outcome variable was analyzed using a Cox proportional hazards model

*SDOH* Social determinants of health, *DBJD* Degenerative bone and joint diseases

### Joint associations of SDOH and lifestyle with DBJDs trajectory

**Fig. 3:**
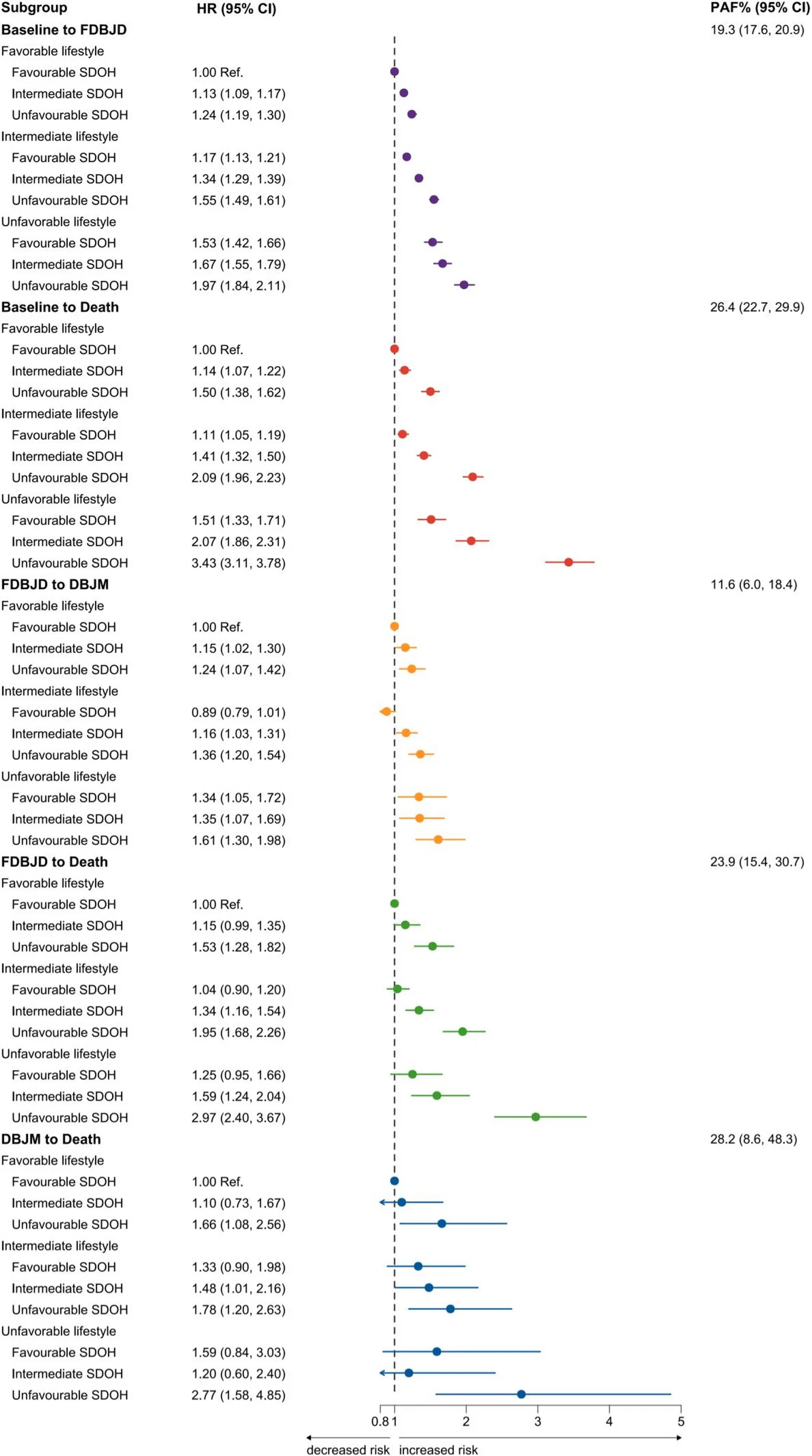
Joint associations of SDOH and lifestyle on risk of the DBJDs trajectory. Models were adjusted for age, sex, history of hypertension, diabetes, and hyperlipidemia. Notes: SDOH, social determinants of health; FDBJD, first degenerative bone and joint diseases; DBJM, degenerative bone and joint multimorbidity; HR, hazard ratio; CI, confidence interval; PAF, population attributable fraction

Figure 3 shows the joint associations of SDOH and lifestyle with DBJDs trajectory. Compared to participants with favourable SDOH and lifestyle, those with unfavourable SDOH and lifestyle had substantially elevated risks across all transitions. The strongest associations were observed for mortality outcomes, with an approximately three-times increased risk of death from baseline (HR = 3.43, 95%CI = 3.11–3.78), as well as death after FDBJD (HR = 2.97, 95%CI = 2.40–3.67) and after DBJM (HR = 2.77, 95%CI = 1.58–4.85). Elevated risks were also observed for disease onset and progression, with HRs of 1.97 (95%CI = 1.84–2.11) for baseline to FDBJD and 1.61 (95%CI = 1.30–1.98) for FDBJD to DBJM. Of note, additive interaction analyses showed significant synergistic effects between SDOH and lifestyle for mortality transitions, particularly death from baseline and death after FDBJD (Table S12), with approximately 28–30% of the excess risk among individuals with joint exposure attributable to interaction. Correspondingly, PAFs for the joint exposure were considerable across all transitions (ranging 11.6%-28.2%), particularly for mortality outcomes.

### Sensitivity analyses

Results remained robust across all sensitivity analyses: excluding participants with missing covariates (Table S13), excluding events occurring within the first 6 months and first year of follow-up (Table S14-S15), excluding participants who entered different states on the same date (Table S16), using different time intervals (1, 30, and 365 days) to define the entry date of the prior state when participants entered different states on the same day (Table S17), using weighted combined SDOH scores (Table S18), adopting different classifications of SDOH (Table S19), excluding “psychosocial problems” and “emotional distress” from the SDOH score (Table S20), and using imputation for all missing variables (Table S21-22),

## Discussion

In this large prospective cohort study, unfavourable SDOH was consistently associated with increased risks across the DBJDs trajectory, with stronger associations observed for mortality transitions than for disease onset. Economic stability and social and community context emerged as the most consistently influential domains, whereas education and healthcare access were mainly related to disease onset, and neighbourhood factors showed little association with progression. Lifestyle explained only a small proportion of the excess risk associated with unfavourable SDOH, and PAF analyses suggested a meaningful model-based attributable fraction under the hypothetical favourable-SDOH reference scenario, particularly for mortality transitions.

Our findings extend and integrate a fragmented body of prior evidence linking adverse social conditions to musculoskeletal outcomes. Previous studies have reported higher mortality risk in adults with osteoarthritis or osteoporosis exposed to social disadvantage [9, 25], poorer clinical outcomes after total knee arthroplasty [10], and elevated secondary fracture risk following initial fragility fractures [26]. However, previous research has largely been confined to single diseases, isolated stages, or individual social indicators, such as socioeconomic status, race/ethnicity, or insurance type [11–16]. By examining multiple social domains simultaneously and across successive disease stages, our study provides a more comprehensive perspective, showing that SDOH act as upstream and systemic determinants shaping progression across the entire DBJD continuum. Furthermore, the particularly strong associations observed for mortality transitions are consistent with evidence that social disadvantage accumulates over time, adversely affecting access healthcare, disease management, and comorbidity burden, thereby amplifying survival disparities [27, 28].

Among individual SDOH domains, economic stability and social and community context showed the most robust and consistent associations across disease transitions. Economic stability reflects access to essential resources, including secure housing, stable employment, adequate income, transportation, and healthy food [29], all of which are fundamental for effective disease management. Economic instability may undermine health through multiple pathways, including reduced access to health care, lower adherence to medications due to cost, and chronic psychosocial stress [12, 13, 29, 30]. Similarly, social and community context, including social capital, social cohesion, and social support, has been linked to musculoskeletal health through both psychological pathways (e.g., depression, and stress-related neuroendocrine activation) and behavioral pathways such as smoking and substance abuse [31–35]. These findings suggest that prioritizing interventions targeting economic security and social support structures, particularly in resource-constrained settings, could yield disproportionate benefits in reducing disparities and improving outcomes across the full course.

By quantifying PAFs across disease stages, our study provides a complementary measure of the potential population-level relevance of social inequalities. Unlike relative risk estimates alone, PAFs incorporate both the strength of association and the exposure distribution in the study population [36, 37]. The larger PAF estimates observed for mortality transitions suggest that social disadvantage may be particularly relevant to survival-related transitions, although these estimates should be interpreted as theoretical, model-based quantities rather than direct estimates of preventable burden.

Although lifestyle factors are commonly targeted in musculoskeletal disease prevention [3–5], we found that they accounted for only a modest proportion of the associations between SDOH and disease progression, with clear stage-specific variation. Mediation by lifestyle was most pronounced at disease onset, when modifiable behaviours act as proximal pathways linking social disadvantages to disease occurrence. Even at this stage, however, lifestyle explained only about 10% of the association. After degenerative disease is established, the mediating role of lifestyle becomes limited. Subsequent progression and survival are increasingly driven by cumulative and structural influences beyond individual behaviors. These upstream factors may promote metabolic dysregulation, chronic inflammation, and functional decline [38], thereby attenuating the relative contribution of lifestyle to later disease transitions. Collectively, the low mediation proportion indicated that meaningful reductions in socially patterned health inequalities are unlikely to be achieved by lifestyle modification alone, underscoring the need for clinical and policy interventions that address upstream social determinants.

The observed joint effects of adverse social determinants and unhealthy lifestyle further emphasize the compounding nature of social and behavioral risk. Individuals exposed to both factors experienced markedly elevated risks, particularly for mortality, with evidence of additive interaction. Such joint effect suggests that social disadvantage and adverse lifestyle do not operate in isolation but rather co-occur and reinforce each other in shaping disease progression and survival [39, 40]. For clinicians, this underscores the importance of integrating social context into risk stratification and management decisions, rather than focusing exclusively on biomedical or behavioral factors. From a population health perspective, this synergy identifies groups for whom integrated interventions addressing both social conditions and health behaviors may yield the greatest reductions in avoidable burden.

To our knowledge, this study was among the first to investigate the associations between SDOH, lifestyle, and the entire DBJDs continuum, from onset through multimorbidity to mortality. The large sample size, long-term follow-up, and consistent results from sensitivity analyses ensured the robustness of findings. Nevertheless, several limitations should be acknowledged. First, information on social determinants and lifestyle was mainly self-reported, which may introduce measurement error and exposure misclassification. Second, single baseline assessment limited the ability to capture the long term SDOH trajectories as well as lifestyle changes during adulthood. Third, differences in baseline characteristics between participants with complete data and those excluded due to missing SDOH data may raise concerns about selection bias. Nevertheless, multiple imputation analyses yielded results largely consistent with the primary findings, suggesting that the influence of missing-data exclusion was limited. Fourth, outcome ascertainment based on hospital admission and death registry data may primarily capture more severe cases of DBJDs, while milder cases managed in outpatient or primary care settings may be under-ascertained. Fifth, although we controlled key personal characteristics and comorbidities, residual confounding cannot be completely ruled out and causal inferences cannot be established in an observational study. Finally, the UKB cohort is predominantly of European ancestry and exhibits a healthy volunteer bias, warranting caution in generalizing these findings to more ethnically and socioeconomically diverse populations.

## Conclusions

In conclusion, SDOH are major contributors to the entire DBJDs continuum, accounting for a substantial and potentially preventable proportion of adverse musculoskeletal outcomes. Lifestyle factors are partially mediated but not fully explained these associations. Reducing the burden of degenerative musculoskeletal diseases in ageing populations would likely require policies and interventions that address social disadvantage alongside traditional behavioral risk factors.

## Supplementary Information

Supplementary Material 1.

## Data Availability

The genetic and phenotypic UK Biobank data are available on application to the UK Biobank to any researcher worldwide.
