# Supplementary material for: Social determinants of health and lifestyle in the progression of degenerative bone and joint diseases: a prospective multi-state analysis
Source: BMC Public Health. 2026 Aug 7;26:3184. doi: 10.1186/s12889-026-28916-7 (PMC13729651; doi:10.1186/s12889-026-28916-7)
Supplement: Supplementary file 1 [file 12889_2026_28916_MOESM1_ESM.docx]

**Social determinants of health and lifestyle in the progression of degenerative bone and joint diseases: a prospective multi-state analysis**

**Table of contents**

**Table S1.** Assessment of social determinants of health in the UK Biobank.

**Table S2.** *β* coefficients for each SDOH component in UK Biobank.

**Table S3.** Assessment of lifestyle factors.

**Table S4.** Codes used to identify outcomes in the UK Biobank.

**Table S5.** The definition and measurements of covariates.

**Table S6.** Different classification choices of SDOH in UK Biobank.

**Table S7.** Associations of social determinants of health with the risk of first degenerative bone and joint diseases using Cox model.

**Table S8.** Associations of social determinants of health with the risk of degenerative bone and joint multimorbidity using Cox model.

**Table S9.** Associations of social determinants of health with the risk of death using Cox model.

**Table S10.** Associations of social determinants of health with different transitions by multi-state model.

**Table S11.** Sensitivity analysis of the mediating role of lifestyle modeled as an ordinal mediator in the association of SDOH with transitions in the DBJDs trajectory.

**Table S12.** Additive interaction between SDOH and lifestyle on risk of the DBJDs trajectory.

**Table S13.** Associations of social determinants of health with the degenerative bone and joint multimorbidity progression, when excluding participants with missing covariates (N = 229,595).

**Table S14.** Associations of social determinants of health with the degenerative bone and joint multimorbidity progression, when excluding outcome events that occurred in the first six months of follow-up (N = 282,246).

**Table S15.** Associations of social determinants of health with the degenerative bone and joint multimorbidity progression, when excluding outcome events that occurred in the first year of follow-up (N = 281,044).

**Table S16.** Associations of social determinants of health with the degenerative bone and joint multimorbidity progression, when excluding participants entering different states at the same date (N = 282,231).

**Table S17.** Associations of social determinants of health with the degenerative bone and joint multimorbidity progression, using different intervals for the participants entering different states at the same date (N = 283,146).

**Table S18.** Associations of social determinants of health with different transitions by multi-state model: using weighted SDOH scores.

**Table S19.** Associations of social determinants of health with different transitions by multi-state model: using different classification of SDOH.

**Table S20.** Associations of social determinants of health with different transitions by multi-state model: using 15 SDOH indicators after excluding psychosocial problems and emotional distress.

**Table S21.** Baseline characteristics of participants included in and excluded from the primary analyses due to missing data after excluding participants with prevalent DBJDs at baseline.

**Table S22.** Associations of social determinants of health with different transitions by multi-state model after multiple imputation of missing SDOH variables and covariates.

| **Table S1. Assessment of social determinants of health in the UK Biobank.** | | | |
| --- | --- | --- | --- |
| **Social determinants of health Domain** | **Advantaged level (0)** | **Disadvantaged level (1)** | **Field ID** |
| **Economic stability** | | | |
| Household income | Average total household income before tax is more than £31,000 | Less than £31,000 | 738; 10877 (pilot) |
| Employment status | Employed, retired or student | Unemployed | 6142 |
| Income quality | Below the median of income deprivation score | Above the median | 26411 (England); 26428 (Scotland); 26418 (Wales) |
| **Education access and quality** | | | |
| Education attainment | Highest education level is college or above | Lower than college | 6138; 10722 (pilot) |
| Education quality | Below the median of education deprivation score | Above the median | 26414 (England); 26431 (Scotland); 26421 (Wales) |
| **Healthcare access and quality** | | | |
| Healthcare | Below the median of healthcare deprivation score | Above the median | 26413 (England); 26430 (Scotland); 26420 (Wales) |
| **Neighborhood and built environment** | | | |
| Accommodation stability | Own home outright | Rent or other arrangement | 680 |
| Housing quality | Below the median of housing deprivation score | Above the median | 26415 (England); 26432 (Scotland); 26423 (Wales) |
| Local crime rate | Below the median of crime score | Above the median | 26416 (England); 26434 (Scotland); 26425 (Wales) |
| Natural environment | Above the median of percentage of home location buffer classed as natural land | Below the median | 24506 |
| **Social and community context** | | | |
| Psychosocial problems | None | With diagnosed psychosocial problems including anxiety, depression | 2100; 2090; 2050; 2060; 2070; 2080; 41270 |
| Race/ethnicity | Black Race | Others | 21000 |
| Living alone/with partners | Living with partners | Living alone | 709 |
| Social support | Able to confide in anyone close to you at least once a week | Once a week or less often | 2110 |
| Social activity | Attend any group activities once a week or more often | Less often than once a week | 6160 |
| Social isolation | Visit friend/family or have them visit you more often than once a week | Once a week or less often | 1031 |
| Emotional distress | None | Have experienced illness, injury, bereavement, stress within last 2 years | 6145 |

| **Table S2. *β* coefficients for each SDOH component in UK Biobank.** | |
| --- | --- |
| **SDOH** | **Adjusted *β* (95% CI)** |
| **Economic stability** |  |
| Household income | 0.35 (0.32, 0.38) |
| Employment status | 0.43 (0.39, 0.47) |
| Income quality | 0.20 (0.17, 0.23) |
| **Education access and quality** |  |
| Education attainment | 0.11 (0.08, 0.14) |
| Education quality | 0.20 (0.17, 0.22) |
| **Healthcare access and quality** |  |
| Healthcare | 0.17 (0.14, 0.19) |
| **Neighborhood and built environment** |  |
| Accommodation stability | 0.10 (0.06, 0.13) |
| Housing quality | 0.01 (-0.02, 0.03) |
| Local crime rate | 0.11 (0.08, 0.14) |
| Natural environment | 0.06 (0.03, 0.09) |
| **Social and community context** |  |
| Psychosocial problems | 0.28 (0.25, 0.31) |
| Race/ethnicity | 0.00 (-0.15, 0.15) |
| Living alone/with partners | 0.34 (0.31, 0.38) |
| Social support | 0.10 (0.07, 0.13) |
| Social activity | 0.08 (0.05, 0.11) |
| Social isolation | 0.01 (-0.02, 0.04) |
| Emotional distress | 0.24 (0.21, 0.27) |
| Model was adjusted for age, sex, smoking status, alcohol consumption, dietary pattern, physical activity, sleep duration, BMI, and history of hypertension, diabetes, and hyperlipidemia. Abbreviations: SDOH, social determinants of health; CI, confidence interval. | |

| **Table S3. Assessment of lifestyle factors.** | | | | |
| --- | --- | --- | --- | --- |
| **Variables** | **Field ID** | **Descriptions** | **Healthy** | **Unhealthy** |
| **Smoking status** | 20116 | Never/previous/current. | non-current smoking | current smoking |
| **Alcohol consumption** | 1558 | Moderate or no drinking (≤ once or twice per week)/heavy drinking (> once or twice per week). | moderate or no drinking | heavy drinking |
| **Healthy diet patterns** | 1289, 1299, 1309, 1319, 1329, 1339, 1349, 1369, 1379, 1389, 1438, 1448, 1458, 1468 | Each diet was scored as healthy (1 point) if processed meats intake (< median), unprocessed meats intake (< median), fish intake (≥ median), vegetable intake (≥ median), fruit intake (≥ median), whole grains (≥ median), and refined grains (< median). The healthy diet scores were summed, ranging from 0 to 7. Healthy diet pattern was defined as healthy diet score ≥ 4. | healthy diet, score ≥ 4 | unhealthy diet, score < 4 |
| **Physical activity** | 22040 | Metabolic equivalent task—sum of minutes performing walking, moderate, and vigorous activity. We categorised participants by three mutually exclusive groups: low (< 600 metabolic equivalent (MET)-min/week), moderate (600 to < 3000 MET-min/week), and vigorous (≥ 3000 MET-min/week) PA based on a standard scoring criteria; the threshold at 600 MET-min/week is equivalent to reaching the recommended guidelines (150 min per week) for moderate-intensity PA. | moderate or vigorous physical activity | low physical activity |
| **Sleep duration** | 1160 | Short: ≤ 6 hours, day; normal: 7 to 8 hours, day; and long: ≥ 9 hours, day. | healthy sleep (7 to 8 hours per night) | unhealthy sleep (≤ 6 or ≥ 9 hours per night) |
| **BMI** | 21001 | Height and weight were measured during the assessment visit, and BMI was calculated as weight in kilograms divided by height in meters squared (kg/m2). | non-obesity  (BMI < 30 kg/m2) | obesity  (BMI ≥ 30 kg/m2) |

| **Table S4. Codes used to identify outcomes in the UK Biobank.** | |
| --- | --- |
| **Outcome** | **ICD codes** |
| **Osteoarthritis** | ICD-10: M15, M150, M1500, M151, M152, M153, M154, M158, M159, M1599, M16, M160, M161, M162, M163, M164, M165, M166, M167, M169, M17, M170, M171, M172, M173, M174, M175, M179, M18, M180, M181, M182, M183, M185, M189, M19, M190, M1900, M1901, M1902, M1903, M1904, M1905, M1906, M1907, M1908, M1909, M191, M1910, M1911, M1912, M1913, M1914, M1915, M1916, M1917, M1918, M1919, M192, M1920, M1921, M1922, M1923, M1924, M1925, M1926, M1927, M1929, M198, M1980, M1981, M1983, M1984, M1985, M1986, M1987, M1988, M1989, M199, M1990, M1991, M1992, M1993, M1994, M1995, M1996, M1997, M1998, M1999 |
|  | ICD-9: 715, 7150, 7151, 71510, 71511, 71512, 71513, 71514, 71515, 71516, 71517, 71518, 71519, 7152, 71520, 71521, 71522, 71523, 71524, 71525, 71526, 71527, 71528, 71529, 7153, 71530, 71531, 71532, 71533, 71534, 71535, 71536, 71537, 71538, 71539, 7158, 7159 |
| **Osteoporosis** | ICD-10: M80, M800, M802, M804, M805, M808, M809, M81, M810, M811, M812, M814, M815, M816, M818, M819, M82, M821 |
|  | ICD-9: 7330, 73300, 73301, 73302, 73303, 73304, 73305, 73306, 73307, 73308, 73309 |
| IVDD | ICD-10: M50, M500, M501, M503, M508, M509, M51, M510, M511, M5116, M5119, M512, M513, M5137, M514, M518, M519 |
|  | ICD-9: 722, 7220, 7221, 7222, 7225, 7226, 7227, 7228, 7229 |
| Abbreviations: IVDD, intervertebral disc degeneration; ICD, International Classification of Disease. | |

| **Table S5. The definition and measurements of covariates.** | | |
| --- | --- | --- |
| **Variables** | **Field ID** | **Descriptions** |
| Age | 21022 | Continuous, year. |
| Sex | 31 | Female/male. |
| Smoking status | 20116 | Never/previous/current. |
| Alcohol consumption | 1558 | Moderate or no drinking/heavy drinking. |
| Dietary pattern | 1289, 1299, 1309, 1319, 1329, 1339, 1349, 1369, 1379, 1389, 1438, 1448, 1458, 1468 | Healthy diet/unhealthy diet. The healthy diet scores were summed, ranging from 0 to 7. Healthy diet pattern was defined as healthy diet score ≥ 4. |
| Physical activity | 22040 | Moderate or vigorous/low, MET minutes/week. |
| Sleep duration | 1160 | Healthy sleep (7 to 8 hours per night)/unhealthy sleep (≤ 6 or ≥ 9 hours per night). |
| BMI | 21001 | Continuous, kg/m2. |
| History of hypertension | 20002, 6150, 41270, 41271 | No/yes. |
| History of diabetes | 20002, 2443, 41270, 41271 | No/yes. |
| History of hyperlipidemia | 20002, 41270, 41271 | No/yes. |

| **Table S6. Different classification choices of SDOH in UK Biobank.** | |
| --- | --- |
| **SDOH** | **Classification** |
| **Economic stability** |  |
| Household income | Greater than 100,000 |
|  | 52,000 to 100,000 |
|  | 31,000 to 51,999 |
|  | 18,000 to 30,999 |
|  | Less than 18,000 |
| Employment status | Employed, retired or student |
|  | Unemployed |
| Income quality | Bottom tertile of area-level income deprivation score |
|  | Middle tertile |
|  | Top tertile |
| **Education access and quality** |  |
| Education attainment | college or above |
|  | High school or equivalent |
|  | Lower than high school |
| Education quality | Bottom tertile of area-level education deprivation score |
|  | Middle tertile |
|  | Top tertile |
| **Healthcare access and quality** |  |
| Healthcare | Bottom tertile of area-level healthcare deprivation score |
|  | Middle tertile |
|  | Top tertile |
| **Neighborhood and built environment** |  |
| Accommodation stability | Own outright |
|  | Own with a mortgage |
|  | Rent accommodation |
| Housing quality | Bottom tertile of area-level housing deprivation score |
|  | Middle tertile |
|  | Top tertile |
| Local crime rate | Bottom tertile of area-level crime score |
|  | Middle tertile |
|  | Top tertile |
| Natural environment | Top tertile of natural environment percentage, buffer 1000m |
|  | Middle tertile |
|  | Bottom tertile |
| **Social and community context** |  |
| Psychosocial problems | None |
|  | With diagnosed Psychosocial problems including anxiety, depression |
| Race/ethnicity | Black |
|  | Others |
| Living alone/with partners | Living with partners |
|  | living alone |
| Social support | Almost daily |
|  | 2-4 times a week |
|  | About once a week |
|  | About once a month |
|  | Once every few months |
|  | Never or almost never |
| Social activity | Attend any group activities once a week or more often |
|  | Less often than once a week |
| Social isolation | Almost daily |
|  | 2-4 times a week |
|  | About once a week |
|  | About once a month |
|  | Once every few months |
|  | Never or almost never |
| Emotional distress | None |
|  | Have experienced illness, injury, bereavement, stress within last 2 years |
| Abbreviations: SDOH, social determinants of health. | |

| **Table S7. Associations of social determinants of health with the risk of first degenerative bone and joint diseases using Cox model.** | | | | | | |
| --- | --- | --- | --- | --- | --- | --- |
|  | **N** | **FDBJD** | | | | |
|  |  | **Cases/Person-years** | **Model 1** | | **Model 2** | |
|  |  |  | **HR (95% CI)** | ***P* value** | **HR (95% CI)** | ***P* value** |
| **Overall SDOH** | | | | | | |
| Favourable [0-5] | 107,773 | 15,609/1,389,312.3 | 1.00 Ref. |  | 1.00 Ref. |  |
| Intermediate [6-8] | 104,024 | 15,894/1,327,879.0 | **1.15 (1.12, 1.17)** | **< 0.001** | **1.09 (1.06, 1.11)** | **< 0.001** |
| Unfavourable [9-17] | 71,349 | 11,669/904,263.5 | **1.36 (1.32, 1.39)** | **< 0.001** | **1.18 (1.15, 1.21)** | **< 0.001** |
| Per score point | 283,146 | 43,172/3,621,454.8 | **1.05 (1.05, 1.05)** | **< 0.001** | **1.03 (1.02, 1.03)** | **< 0.001** |
| **Individual SDOH** | | | | | | |
| **Economic stability** | | | | | | |
| Favourable [0-1] | 213,012 | 30,156/2,740,837.7 | 1.00 Ref. |  | 1.00 Ref. |  |
| Unfavourable [2-3] | 70,134 | 13,016/880,617.1 | **1.12 (1.09, 1.14)** | **< 0.001** | **1.08 (1.05, 1.10)** | **< 0.001** |
| **Education access and quality** | | | | | | |
| Favourable [0-1] | 209,715 | 31,269/2,684,765.7 | 1.00 Ref. |  | 1.00 Ref. |  |
| Unfavourable [2] | 73,431 | 11,903/936,689.1 | **1.10 (1.08, 1.13)** | **< 0.001** | **1.04 (1.02, 1.07)** | **< 0.001** |
| **Healthcare access and quality** | | | | | | |
| Favourable [0] | 154,514 | 22,347/1,980,660.6 | 1.00 Ref. |  | 1.00 Ref. |  |
| Unfavourable [1] | 128,632 | 20,825/1,640,794.2 | **1.13 (1.10, 1.15)** | **< 0.001** | **1.09 (1.07, 1.11)** | **< 0.001** |
| **Neighborhood and built environment** | | | | | | |
| Favourable [0-2] | 188,164 | 30,199/2,406,615.4 | 1.00 Ref. |  | 1.00 Ref. |  |
| Unfavourable [3-4] | 94,982 | 12,973/1,214,839.4 | **0.95 (0.93, 0.97)** | **< 0.001** | **0.93 (0.91, 0.95)** | **< 0.001** |
| **Social and community context** | | | | | | |
| Favourable [0-2] | 166,739 | 24,423/2,142,477.9 | 1.00 Ref. |  | 1.00 Ref. |  |
| Unfavourable [3-7] | 116,407 | 18,749/1,478,976.8 | **1.15 (1.13, 1.18)** | **< 0.001** | **1.10 (1.08, 1.12)** | **< 0.001** |
| Model 1 was adjusted for age and sex. Model 2 was further adjusted for smoking status, alcohol consumption, dietary pattern, physical activity, sleep duration, BMI, history of hypertension, diabetes, and hyperlipidemia. For analyses of individual social determinants of health factors, all social determinants of health factors were simultaneously adjustesd. Notes: SDOH, social determinants of health; FDBJD, first degenerative bone and joint diseases; BMI, body mass index; HR, hazard ratio; CI, confidence interval. | | | | | | |

| **Table S8. Associations of social determinants of health with the risk of degenerative bone and joint multimorbidity using Cox model.** | | | | | | |
| --- | --- | --- | --- | --- | --- | --- |
|  | **N** | **DBJM** | | | | |
|  |  | **Cases/Person-years** | **Model 1** | | **Model 2** | |
|  |  |  | **HR (95% CI)** | ***P* value** | **HR (95% CI)** | ***P* value** |
| **Overall SDOH** | | | | | | |
| Favourable [0-5] | 107,773 | 1,341/1,477,980.8 | 1.00 Ref. |  | 1.00 Ref. |  |
| Intermediate [6-8] | 104,024 | 1,553/1,416,837.4 | **1.32 (1.23, 1.42)** | **< 0.001** | **1.23 (1.14, 1.32)** | **< 0.001** |
| Unfavourable [9-17] | 71,349 | 1,276/970,194.0 | **1.79 (1.66, 1.94)** | **< 0.001** | **1.49 (1.37, 1.61)** | **< 0.001** |
| Per score point | 283,146 | 4,170/3,865,012.2 | **1.10 (1.09, 1.11)** | **< 0.001** | **1.07 (1.06, 1.08)** | **< 0.001** |
| **Individual SDOH** | | | | | | |
| **Economic stability** | | | | | | |
| Favourable [0-1] | 213,012 | 2,663/2,911,295.8 | 1.00 Ref. |  | 1.00 Ref. |  |
| Unfavourable [2-3] | 70,134 | 1,507/953,716.4 | **1.29 (1.20, 1.39)** | **< 0.001** | **1.22 (1.13, 1.31)** | **< 0.001** |
| **Education access and quality** | | | | | | |
| Favourable [0-1] | 209,715 | 3,001/2,860,790.3 | 1.00 Ref. |  | 1.00 Ref. |  |
| Unfavourable [2] | 73,431 | 1,169/1,004,221.9 | 1.07 (0.99, 1.15) | 0.092 | 1.01 (0.93, 1.08) | 0.866 |
| **Healthcare access and quality** | | | | | | |
| Favourable [0] | 154,514 | 2,063/2,106,390.9 | 1.00 Ref. |  | 1.00 Ref. |  |
| Unfavourable [1] | 128,632 | 2,107/1,758,621.3 | **1.17 (1.09, 1.25)** | **< 0.001** | **1.13 (1.05, 1.21)** | **0.001** |
| **Neighborhood and built environment** | | | | | | |
| Favourable [0-2] | 188,164 | 2,928/2,577,226.4 | 1.00 Ref. |  | 1.00 Ref. |  |
| Unfavourable [3-4] | 94,982 | 1,242/1,287,785.8 | 0.97 (0.91, 1.04) | 0.452 | 0.94 (0.88, 1.01) | 0.082 |
| **Social and community context** | | | | | | |
| Favourable [0-2] | 166,739 | 2,138/2,280,768.0 | 1.00 Ref. |  | 1.00 Ref. |  |
| Unfavourable [3-7] | 116,407 | 2,032/1,584,244.2 | **1.41 (1.33, 1.50)** | **< 0.001** | **1.30 (1.22, 1.39)** | **< 0.001** |
| Model 1 was adjusted for age and sex. Model 2 was further adjusted for smoking status, alcohol consumption, dietary pattern, physical activity, sleep duration, BMI, history of hypertension, diabetes, and hyperlipidemia. For analyses of individual social determinants of health factors, all social determinants of health factors were simultaneously adjustesd. Notes: SDOH, social determinants of health; DBJM, degenerative bone and joint multimorbidity; BMI, body mass index; HR, hazard ratio; CI, confidence interval. | | | | | | |

| **Table S9. Associations of social determinants of health with the risk of death using Cox model.** | | | | | | |
| --- | --- | --- | --- | --- | --- | --- |
|  | **N** | **Death** | | | | |
|  |  | **Cases/Person-years** | **Model 1** | | **Model 2** | |
|  |  |  | **HR (95% CI)** | ***P* value** | **HR (95% CI)** | ***P* value** |
| **Overall SDOH** | | | | | | |
| Favourable [0-5] | 107,773 | 6,417/1,451,122.8 | 1.00 Ref. |  | 1.00 Ref. |  |
| Intermediate [6-8] | 104,024 | 6,570/1,388,897.5 | **1.27 (1.23, 1.32)** | **< 0.001** | **1.17 (1.13, 1.21)** | **< 0.001** |
| Unfavourable [9-17] | 71,349 | 5,844/944,709.2 | **2.01 (1.94, 2.09)** | **< 0.001** | **1.62 (1.56, 1.68)** | **< 0.001** |
| Per score point | 283,146 | 18,831/3,784,729.5 | **1.12 (1.12, 1.13)** | **< 0.001** | **1.08 (1.08, 1.09)** | **< 0.001** |
| **Individual SDOH** | | | | | | |
| **Economic stability** | | | | | | |
| Favourable [0-1] | 213,012 | 11,902/2,860,984.5 | 1.00 Ref. |  | 1.00 Ref. |  |
| Unfavourable [2-3] | 70,134 | 6,929/923,745.1 | **1.46 (1.41, 1.51)** | **< 0.001** | **1.35 (1.31, 1.40)** | **< 0.001** |
| **Education access and quality** | | | | | | |
| Favourable [0-1] | 209,715 | 13,237/2,805,068.2 | 1.00 Ref. |  | 1.00 Ref. |  |
| Unfavourable [2] | 73,431 | 5,594/979,661.3 | **1.18 (1.14, 1.22)** | **< 0.001** | **1.10 (1.07, 1.14)** | **< 0.001** |
| **Healthcare access and quality** | | | | | | |
| Favourable [0] | 154,514 | 9,560/2,065,881.8 | 1.00 Ref. |  | 1.00 Ref. |  |
| Unfavourable [1] | 128,632 | 9,271/1,718,847.7 | **1.04 (1.01, 1.08)** | **0.018** | 1.00 (0.97, 1.04) | 0.773 |
| **Neighborhood and built environment** | | | | | | |
| Favourable [0-2] | 188,164 | 13,116/2,522,051.2 | 1.00 Ref. |  | 1.00 Ref. |  |
| Unfavourable [3-4] | 94,982 | 5,715/1,262,678.3 | **1.08 (1.04, 1.11)** | **< 0.001** | 1.03 (0.99, 1.06) | 0.131 |
| **Social and community context** | | | | | | |
| Favourable [0-2] | 166,739 | 10,044/2,238,775.0 | 1.00 Ref. |  | 1.00 Ref. |  |
| Unfavourable [3-7] | 116,407 | 8,787/1,545,954.6 | **1.38 (1.34, 1.43)** | **< 0.001** | **1.27 (1.23, 1.31)** | **< 0.001** |
| Model 1 was adjusted for age and sex. Model 2 was further adjusted for smoking status, alcohol consumption, dietary pattern, physical activity, sleep duration, BMI, history of hypertension, diabetes, and hyperlipidemia. For analyses of individual social determinants of health factors, all social determinants of health factors were simultaneously adjustesd. Notes: SDOH, social determinants of health; BMI, body mass index; HR, hazard ratio; CI, confidence interval. | | | | | | |

| **Table S10. Associations of social determinants of health with different transitions by multi-state model.** | | | | | | | | | | |
| --- | --- | --- | --- | --- | --- | --- | --- | --- | --- | --- |
| **Overall SDOH** | **Baseline to FDBJD** | | **Baseline to Death** | | **FDBJD to DBJM** | | **FDBJD to Death** | | **DBJM to Death** | |
|  | **HR (95% CI)** | ***P* value** | **HR (95% CI)** | ***P* value** | **HR (95% CI)** | ***P* value** | **HR (95% CI)** | ***P* value** | **HR (95% CI)** | ***P* value** |
| Favourable [0-5] | 1.00 Ref. |  | 1.00 Ref. |  | 1.00 Ref. |  | 1.00 Ref. |  | 1.00 Ref. |  |
| Intermediate [6-8] | **1.10 (1.07, 1.12)** | **< 0.001** | **1.16 (1.12, 1.21)** | **< 0.001** | **1.17 (1.09, 1.26)** | **< 0.001** | **1.19 (1.10, 1.30)** | **< 0.001** | 1.10 (0.88, 1.38) | 0.421 |
| Unfavourable [9-17] | **1.21 (1.18, 1.24)** | **< 0.001** | **1.58 (1.52, 1.65)** | **< 0.001** | **1.33 (1.23, 1.44)** | **< 0.001** | **1.64 (1.50, 1.80)** | **< 0.001** | **1.48 (1.17, 1.87)** | **0.001** |
| Per score point | **1.03 (1.03, 1.03)** | **< 0.001** | **1.08 (1.07, 1.09)** | **< 0.001** | **1.05 (1.04, 1.06)** | **< 0.001** | **1.09 (1.07, 1.10)** | **< 0.001** | **1.07 (1.04, 1.11)** | **< 0.001** |
| Models were adjusted for age, sex, smoking status, alcohol consumption, dietary pattern, physical activity, sleep duration, BMI, history of hypertension, diabetes, and hyperlipidemia. Notes: SDOH, social determinants of health; FDBJD, first degenerative bone and joint diseases; DBJM, degenerative bone and joint multimorbidity; BMI, body mass index; HR, hazard ratio; CI, confidence interval. | | | | | | | | | | |

| **Table S11. Sensitivity analysis of the mediating role of lifestyle modeled as an ordinal mediator in the association of SDOH with transitions in the DBJDs trajectory.** | |
| --- | --- |
| **Transitions** | **Proportion Mediated, (95% CI)** |
| Baseline to FDBJD | 8.69% (7.47%, 9.86%) |
| Baseline to Death | 5.85% (4.77%, 6.64%) |
| FDBJD to DBJM | 1.96% (-7.57%, 4.03%) |
| FDBJD to Death | 4.96% (2.60%, 6.37%) |
| DBJM to Death | 5.48% (0.40%, 28.67%) |
| Models were adjusted for age, sex, history of hypertension, diabetes, and hyperlipidemia. SDOH was the exposure, lifestyle was the mediator, and transition occurrence was the outcome. SDOH was modeled as a standardized continuous variable per 1-SD increase, whereas lifestyle was modeled as an ordinal mediator based on the predefined lifestyle categories. The exposure-mediator model was fitted using ordinal logistic regression, and the mediator-outcome model was fitted using Cox proportional hazards regression. Abbreviations: CI, confidence interval; SDOH, social determinants of health; DBJD, degenerative bone and joint diseases; FDBJD, first degenerative bone and joint disease; DBJM, degenerative bone and joint multimorbidity. | |

| **Table S12. Additive interaction between SDOH and lifestyle on risk of the DBJDs trajectory.** | | | | | |
| --- | --- | --- | --- | --- | --- |
|  | **Baseline to FDBJD** | **Baseline to Death** | **FDBJD to DBJM** | **FDBJD to Death** | **DBJM to Death** |
| *RERI* (95% CI) | 0.09 (-0.04, 0.22) | **0.81 (0.53, 1.09)** | 0.00 (-0.36, 0.36) | **0.77 (0.25, 1.30)** | 0.71 (-0.46, 1.87) |
| *AP* (95% CI) | 0.05 (-0.02, 0.13) | **0.28 (0.20, 0.35)** | 0.00 (-0.24, 0.24) | **0.30 (0.14, 0.45)** | 0.32 (-0.09, 0.72) |
| *S* (95% CI) | 1.15 (0.94, 1.40) | **1.73 (1.45, 2.06)** | 1.00 (0.50, 2.00) | **1.93 (1.27, 2.95)** | 2.39 (0.58, 9.85) |
| Models were adjusted for age, sex, history of hypertension, diabetes, and hyperlipidemia. SDOH and lifestyle were categorized into two groups for additive interaction analysis: intermediate and favorable levels were combined as one group, and unfavorable as the other. Notes: SDOH, social determinants of health; FDBJD, first degenerative bone and joint diseases; DBJM, degenerative bone and joint multimorbidity; RERI, relative excess risk due to interaction; AP, attributable proportion; S, synergy index; CI, confidence interval. | | | | | |

| **Table S13. Associations of social determinants of health with the degenerative bone and joint multimorbidity progression, when excluding participants with missing covariates (N = 229,595).** | | | | | | | | | | |
| --- | --- | --- | --- | --- | --- | --- | --- | --- | --- | --- |
| **Overall SDOH** | **Baseline to FDBJD** | | **Baseline to Death** | | **FDBJD to DBJM** | | **FDBJD to Death** | | **DBJM to Death** | |
|  | **HR (95% CI)** | ***P* value** | **HR (95% CI)** | ***P* value** | **HR (95% CI)** | ***P* value** | **HR (95% CI)** | ***P* value** | **HR (95% CI)** | ***P* value** |
| Favourable [0-5] | 1.00 Ref. |  | 1.00 Ref. |  | 1.00 Ref. |  | 1.00 Ref. |  | 1.00 Ref. |  |
| Intermediate [6-8] | **1.10 (1.07, 1.13)** | **< 0.001** | **1.17 (1.12, 1.22)** | **< 0.001** | **1.20 (1.10, 1.30)** | **< 0.001** | **1.20 (1.09, 1.32)** | **< 0.001** | 1.07 (0.82, 1.38) | 0.630 |
| Unfavourable [9-17] | **1.20 (1.16, 1.23)** | **< 0.001** | **1.58 (1.51, 1.66)** | **< 0.001** | **1.33 (1.21, 1.46)** | **< 0.001** | **1.65 (1.48, 1.83)** | **< 0.001** | **1.47 (1.12, 1.92)** | **0.005** |
| Per score point | **1.03 (1.02, 1.03)** | **< 0.001** | **1.08 (1.07, 1.08)** | **< 0.001** | **1.05 (1.04, 1.07)** | **< 0.001** | **1.09 (1.07, 1.10)** | **< 0.001** | **1.07 (1.03, 1.11)** | **0.001** |
| Models were adjusted for age, sex, smoking status, alcohol consumption, dietary pattern, physical activity, sleep duration, BMI, history of hypertension, diabetes, and hyperlipidemia. Notes: SDOH, social determinants of health; FDBJD, first degenerative bone and joint diseases; DBJM, degenerative bone and joint multimorbidity; BMI, body mass index; HR, hazard ratio; CI, confidence interval. | | | | | | | | | | |

| **Table S14. Associations of social determinants of health with the degenerative bone and joint multimorbidity progression, when excluding outcome events that occurred in the first six months of follow-up (N = 282,246).** | | | | | | | | | | |
| --- | --- | --- | --- | --- | --- | --- | --- | --- | --- | --- |
| **Overall SDOH** | **Baseline to FDBJD** | | **Baseline to Death** | | **FDBJD to DBJM** | | **FDBJD to Death** | | **DBJM to Death** | |
|  | **HR (95% CI)** | ***P* value** | **HR (95% CI)** | ***P* value** | **HR (95% CI)** | ***P* value** | **HR (95% CI)** | ***P* value** | **HR (95% CI)** | ***P* value** |
| Favourable [0-5] | 1.00 Ref. |  | 1.00 Ref. |  | 1.00 Ref. |  | 1.00 Ref. |  | 1.00 Ref. |  |
| Intermediate [6-8] | **1.10 (1.07, 1.12)** | **< 0.001** | **1.16 (1.11, 1.20)** | **< 0.001** | **1.17 (1.08, 1.26)** | **< 0.001** | **1.18 (1.08, 1.29)** | **< 0.001** | 1.10 (0.88, 1.38) | 0.410 |
| Unfavourable [9-17] | **1.21 (1.18, 1.24)** | **< 0.001** | **1.58 (1.51, 1.64)** | **< 0.001** | **1.33 (1.23, 1.45)** | **< 0.001** | **1.64 (1.49, 1.80)** | **< 0.001** | **1.47 (1.16, 1.86)** | **0.002** |
| Per score point | **1.03 (1.03, 1.03)** | **< 0.001** | **1.08 (1.07, 1.09)** | **< 0.001** | **1.05 (1.04, 1.06)** | **< 0.001** | **1.09 (1.07, 1.10)** | **< 0.001** | **1.07 (1.03, 1.11)** | **<0.001** |
| Models were adjusted for age, sex, smoking status, alcohol consumption, dietary pattern, physical activity, sleep duration, BMI, history of hypertension, diabetes, and hyperlipidemia. Notes: SDOH, social determinants of health; FDBJD, first degenerative bone and joint diseases; DBJM, degenerative bone and joint multimorbidity; BMI, body mass index; HR, hazard ratio; CI, confidence interval. | | | | | | | | | | |

| **Table S15. Associations of social determinants of health with the degenerative bone and joint multimorbidity progression, when excluding outcome events that occurred in the first year of follow-up (N = 281,044).** | | | | | | | | | | |
| --- | --- | --- | --- | --- | --- | --- | --- | --- | --- | --- |
| **Overall SDOH** | **Baseline to FDBJD** | | **Baseline to Death** | | **FDBJD to DBJM** | | **FDBJD to Death** | | **DBJM to Death** | |
|  | **HR (95% CI)** | ***P* value** | **HR (95% CI)** | ***P* value** | **HR (95% CI)** | ***P* value** | **HR (95% CI)** | ***P* value** | **HR (95% CI)** | ***P* value** |
| Favourable [0-5] | 1.00 Ref. |  | 1.00 Ref. |  | 1.00 Ref. |  | 1.00 Ref. |  | 1.00 Ref. |  |
| Intermediate [6-8] | **1.10 (1.07, 1.12)** | **< 0.001** | **1.15 (1.11, 1.20)** | **< 0.001** | **1.17 (1.09, 1.26)** | **< 0.001** | **1.18 (1.08, 1.29)** | **< 0.001** | 1.09 (0.87, 1.38) | 0.446 |
| Unfavourable [9-17] | **1.21 (1.18, 1.24)** | **< 0.001** | **1.57 (1.50, 1.64)** | **< 0.001** | **1.33 (1.23, 1.45)** | **< 0.001** | **1.62 (1.47, 1.78)** | **< 0.001** | **1.47 (1.16, 1.87)** | **0.002** |
| Per score point | **1.03 (1.03, 1.03)** | **< 0.001** | **1.08 (1.07, 1.08)** | **< 0.001** | **1.05 (1.04, 1.06)** | **< 0.001** | **1.08 (1.07, 1.10)** | **< 0.001** | **1.07 (1.03, 1.10)** | **<0.001** |
| Models were adjusted for age, sex, smoking status, alcohol consumption, dietary pattern, physical activity, sleep duration, BMI, history of hypertension, diabetes, and hyperlipidemia. Notes: SDOH, social determinants of health; FDBJD, first degenerative bone and joint diseases; DBJM, degenerative bone and joint multimorbidity; BMI, body mass index; HR, hazard ratio; CI, confidence interval. | | | | | | | | | | |

| **Table S16. Associations of social determinants of health with the degenerative bone and joint multimorbidity progression, when excluding participants entering different states at the same date (N = 282,231).** | | | | | | | | | | |
| --- | --- | --- | --- | --- | --- | --- | --- | --- | --- | --- |
| **Overall SDOH** | **Baseline to FDBJD** | | **Baseline to Death** | | **FDBJD to DBJM** | | **FDBJD to Death** | | **DBJM to Death** | |
|  | **HR (95% CI)** | ***P* value** | **HR (95% CI)** | ***P* value** | **HR (95% CI)** | ***P* value** | **HR (95% CI)** | ***P* value** | **HR (95% CI)** | ***P* value** |
| Favourable [0-5] | 1.00 Ref. |  | 1.00 Ref. |  | 1.00 Ref. |  | 1.00 Ref. |  | 1.00 Ref. |  |
| Intermediate [6-8] | **1.09 (1.07, 1.11)** | **< 0.001** | **1.16 (1.12, 1.21)** | **< 0.001** | **1.13 (1.03, 1.23)** | **0.006** | **1.20 (1.10, 1.30)** | **< 0.001** | 1.02 (0.80, 1.31) | 0.875 |
| Unfavourable [9-17] | **1.20 (1.17, 1.23)** | **< 0.001** | **1.59 (1.52, 1.65)** | **< 0.001** | **1.37 (1.25, 1.50)** | **< 0.001** | **1.64 (1.50, 1.80)** | **< 0.001** | **1.37 (1.06, 1.76)** | **0.016** |
| Per score point | **1.03 (1.03, 1.03)** | **< 0.001** | **1.08 (1.07, 1.09)** | **< 0.001** | **1.05 (1.04, 1.07)** | **< 0.001** | **1.09 (1.07, 1.10)** | **< 0.001** | **1.06 (1.02, 1.10)** | **0.001** |
| Models were adjusted for age, sex, smoking status, alcohol consumption, dietary pattern, physical activity, sleep duration, BMI, history of hypertension, diabetes, and hyperlipidemia. Notes: SDOH, social determinants of health; FDBJD, first degenerative bone and joint diseases; DBJM, degenerative bone and joint multimorbidity; BMI, body mass index; HR, hazard ratio; CI, confidence interval. | | | | | | | | | | |

| **Table S17. Associations of social determinants of health with the degenerative bone and joint multimorbidity progression, using different intervals for the participants entering different states at the same date (N = 283,146).** | | | | | | | | | | |
| --- | --- | --- | --- | --- | --- | --- | --- | --- | --- | --- |
| **Overall SDOH** | **Baseline to FDBJD** | | **Baseline to Death** | | **FDBJD to DBJM** | | **FDBJD to Death** | | **DBJM to Death** | |
|  | **HR (95% CI)** | ***P* value** | **HR (95% CI)** | ***P* value** | **HR (95% CI)** | ***P* value** | **HR (95% CI)** | ***P* value** | **HR (95% CI)** | ***P* value** |
| **1 day** |  |  |  |  |  |  |  |  |  |  |
| Favourable [0-5] | 1.00 Ref. |  | 1.00 Ref. |  | 1.00 Ref. |  | 1.00 Ref. |  | 1.00 Ref. |  |
| Intermediate [6-8] | **1.10 [1.07, 1.12]** | **< 0.001** | **1.16 (1.12, 1.21)** | **< 0.001** | **1.17 [1.09, 1.26]** | **< 0.001** | **1.19 [1.10, 1.30]** | **< 0.001** | 1.10 [0.88, 1.38] | 0.421 |
| Unfavourable [9-17] | **1.21 [1.18, 1.24]** | **< 0.001** | **1.58 [1.52, 1.65]** | **< 0.001** | **1.33 [1.23, 1.44]** | **< 0.001** | **1.64 (1.50, 1.80)** | **< 0.001** | **1.48 [1.17, 1.87]** | **0.001** |
| Per score point | **1.03 (1.03, 1.03)** | **< 0.001** | **1.08 (1.07, 1.09)** | **< 0.001** | **1.05 [1.04, 1.06]** | **< 0.001** | **1.09 (1.07, 1.10)** | **< 0.001** | **1.07 [1.04, 1.11]** | **< 0.001** |
| **30 days** |  |  |  |  |  |  |  |  |  |  |
| Favourable [0-5] | 1.00 Ref. |  | 1.00 Ref. |  | 1.00 Ref. |  | 1.00 Ref. |  | 1.00 Ref. |  |
| Intermediate [6-8] | **1.10 [1.07, 1.12]** | **< 0.001** | **1.16 (1.12, 1.21)** | **< 0.001** | **1.17 [1.09, 1.26]** | **< 0.001** | **1.19 [1.10, 1.30]** | **< 0.001** | 1.10 [0.88, 1.38] | 0.421 |
| Unfavourable [9-17] | **1.21 [1.18, 1.24]** | **< 0.001** | **1.58 [1.52, 1.65]** | **< 0.001** | **1.33 [1.23, 1.44]** | **< 0.001** | **1.64 (1.50, 1.80)** | **< 0.001** | **1.48 [1.17, 1.87]** | **0.001** |
| Per score point | **1.03 (1.03, 1.03)** | **< 0.001** | **1.08 (1.07, 1.09)** | **< 0.001** | **1.05 (1.04, 1.06)** | **< 0.001** | **1.09 (1.07, 1.10)** | **< 0.001** | **1.07 [1.04, 1.11]** | **< 0.001** |
| **365 days** |  |  |  |  |  |  |  |  |  |  |
| Favourable [0-5] | 1.00 Ref. |  | 1.00 Ref. |  | 1.00 Ref. |  | 1.00 Ref. |  | 1.00 Ref. |  |
| Intermediate [6-8] | **1.10 [1.07, 1.12]** | **< 0.001** | **1.16 (1.12, 1.21)** | **< 0.001** | **1.17 [1.08, 1.26]** | **< 0.001** | **1.19 [1.09, 1.30]** | **< 0.001** | 1.09 [0.87, 1.37] | 0.442 |
| Unfavourable [9-17] | **1.21 [1.18, 1.24]** | **< 0.001** | **1.58 [1.52, 1.65]** | **< 0.001** | **1.33 [1.23, 1.44]** | **< 0.001** | **1.64 (1.49, 1.80)** | **< 0.001** | **1.49 [1.18, 1.89]** | **0.001** |
| Per score point | **1.03 (1.03, 1.03)** | **< 0.001** | **1.08 (1.07, 1.09)** | **< 0.001** | **1.05 (1.04, 1.06)** | **< 0.001** | **1.09 (1.07, 1.10)** | **< 0.001** | **1.07 [1.04, 1.11]** | **< 0.001** |
| Models were adjusted for age, sex, smoking status, alcohol consumption, dietary pattern, physical activity, sleep duration, BMI, history of hypertension, diabetes, and hyperlipidemia. Notes: SDOH, social determinants of health; FDBJD, first degenerative bone and joint diseases; DBJM, degenerative bone and joint multimorbidity; BMI, body mass index; HR, hazard ratio; CI, confidence interval. | | | | | | | | | | |

| **Table S18. Associations of social determinants of health with different transitions by multi-state model: using weighted SDOH scores.** | | | | | | | | | | |
| --- | --- | --- | --- | --- | --- | --- | --- | --- | --- | --- |
| **Overall SDOH** | **Baseline to FDBJD** | | **Baseline to Death** | | **FDBJD to DBJM** | | **FDBJD to Death** | | **DBJM to Death** | |
|  | **HR (95% CI)** | ***P* value** | **HR (95% CI)** | ***P* value** | **HR (95% CI)** | ***P* value** | **HR (95% CI)** | ***P* value** | **HR (95% CI)** | ***P* value** |
| Favourable [0-5] | 1.00 Ref. |  | 1.00 Ref. |  | 1.00 Ref. |  | 1.00 Ref. |  | 1.00 Ref. |  |
| Intermediate [6-8] | **1.15 (1.12, 1.17)** | **< 0.001** | **1.20 (1.15, 1.26)** | **< 0.001** | **1.25 (1.15, 1.37)** | **< 0.001** | **1.23 (1.11, 1.35)** | **< 0.001** | 0.93 (0.71, 1.21) | 0.578 |
| Unfavourable [9-17] | **1.31 (1.28, 1.35)** | **< 0.001** | **1.63 (1.57, 1.70)** | **< 0.001** | **1.50 (1.38, 1.63)** | **< 0.001** | **1.67 (1.52, 1.84)** | **< 0.001** | **1.32 (1.03, 1.69)** | **0.031** |
| Per score point | **1.04 (1.04, 1.04)** | **< 0.001** | **1.08 (1.08, 1.09)** | **< 0.001** | **1.05 (1.04, 1.06)** | **< 0.001** | **1.08 (1.07, 1.09)** | **< 0.001** | **1.06 (1.03, 1.09)** | **< 0.001** |
| Models were adjusted for age, sex, smoking status, alcohol consumption, dietary pattern, physical activity, sleep duration, BMI, history of hypertension, diabetes, and hyperlipidemia. Notes: SDOH, social determinants of health; FDBJD, first degenerative bone and joint diseases; DBJM, degenerative bone and joint multimorbidity; BMI, body mass index; HR, hazard ratio; CI, confidence interval. | | | | | | | | | | |

| **Table S19. Associations of social determinants of health with different transitions by multi-state model: using different classification of SDOH.** | | | | | | | | | | |
| --- | --- | --- | --- | --- | --- | --- | --- | --- | --- | --- |
| **Overall SDOH** | **Baseline to FDBJD** | | **Baseline to Death** | | **FDBJD to DBJM** | | **FDBJD to Death** | | **DBJM to Death** | |
|  | **HR (95% CI)** | ***P* value** | **HR (95% CI)** | ***P* value** | **HR (95% CI)** | ***P* value** | **HR (95% CI)** | ***P* value** | **HR (95% CI)** | ***P* value** |
| Favourable [0-5] | 1.00 Ref. |  | 1.00 Ref. |  | 1.00 Ref. |  | 1.00 Ref. |  | 1.00 Ref. |  |
| Intermediate [6-8] | **1.12 (1.09, 1.14)** | **< 0.001** | **1.20 (1.16, 1.25)** | **< 0.001** | **1.25 (1.15, 1.35)** | **< 0.001** | **1.21 (1.10, 1.32)** | **< 0.001** | 1.11 (0.86, 1.42) | 0.416 |
| Unfavourable [9-17] | **1.27 (1.23, 1.30)** | **< 0.001** | **1.66 (1.59, 1.73)** | **< 0.001** | **1.48 (1.36, 1.61)** | **< 0.001** | **1.68 (1.53, 1.85)** | **< 0.001** | **1.46 (1.14, 1.88)** | **0.003** |
| Per score point | **1.04 (1.04, 1.05)** | **< 0.001** | **1.11 (1.10, 1.11)** | **< 0.001** | **1.06 (1.05, 1.08)** | **< 0.001** | **1.11 (1.09, 1.12)** | **< 0.001** | **1.07 (1.04, 1.10)** | **< 0.001** |
| Models were adjusted for age, sex, smoking status, alcohol consumption, dietary pattern, physical activity, sleep duration, BMI, history of hypertension, diabetes, and hyperlipidemia. Notes: SDOH, social determinants of health; FDBJD, first degenerative bone and joint diseases; DBJM, degenerative bone and joint multimorbidity; BMI, body mass index; HR, hazard ratio; CI, confidence interval. | | | | | | | | | | |

| **Table S20. Associations of social determinants of health with different transitions by multi-state model: using 15 SDOH indicators after excluding psychosocial problems and emotional distress.** | | | | | | | | | | |
| --- | --- | --- | --- | --- | --- | --- | --- | --- | --- | --- |
| **Overall SDOH** | **Baseline to FDBJD** | | **Baseline to Death** | | **FDBJD to DBJM** | | **FDBJD to Death** | | **DBJM to Death** | |
|  | **HR (95% CI)** | ***P* value** | **HR (95% CI)** | ***P* value** | **HR (95% CI)** | ***P* value** | **HR (95% CI)** | ***P* value** | **HR (95% CI)** | ***P* value** |
| Favourable [0-5] | 1.00 Ref. |  | 1.00 Ref. |  | 1.00 Ref. |  | 1.00 Ref. |  | 1.00 Ref. |  |
| Intermediate [6-8] | **1.05 (1.02, 1.07)** | **< 0.001** | **1.09 (1.05, 1.13)** | **< 0.001** | **1.11 (1.03, 1.19)** | **0.005** | **1.15 (1.05, 1.25)** | **0.002** | 1.19 (0.95, 1.48) | 0.132 |
| Unfavourable [9-17] | **1.09 (1.06, 1.11)** | **< 0.001** | **1.45 (1.39, 1.51)** | **< 0.001** | **1.24 (1.15, 1.35)** | **< 0.001** | **1.59 (1.45, 1.74)** | **< 0.001** | **1.75 (1.39, 2.21)** | **< 0.001** |
| Per score point | **1.01 (1.01, 1.02)** | **< 0.001** | **1.07 (1.06, 1.08)** | **< 0.001** | **1.04 (1.03, 1.05)** | **< 0.001** | **1.08 (1.07, 1.10)** | **< 0.001** | **1.09 (1.05, 1.13)** | **< 0.001** |
| Models were adjusted for age, sex, smoking status, alcohol consumption, dietary pattern, physical activity, sleep duration, BMI, history of hypertension, diabetes, and hyperlipidemia. Notes: SDOH, social determinants of health; FDBJD, first degenerative bone and joint diseases; DBJM, degenerative bone and joint multimorbidity; BMI, body mass index; HR, hazard ratio; CI, confidence interval. | | | | | | | | | | |

| **Table S21. Baseline characteristics of participants included in and excluded from the primary analyses due to missing data after excluding participants with prevalent DBJDs at baseline.** | | | |
| --- | --- | --- | --- |
| **Baseline characteristics** | **Total** | **Included** | **Excluded** |
| **No. of participants** | 438,506 | 283,146 | 155,360 |
| **Age, years** | 56.0 ± 8.1 | 54.9 ± 8.0 | 57.9 ± 8.0 |
| **Female, n (%)** | 232,671 (53.1) | 147,899 (52.2) | 84,772 (54.6) |
| **Smoking status, n (%)** |  |  |  |
| Never | 241,673 (55.1) | 160,876 (56.8) | 80,797 (52.0) |
| Previous | 148,082 (33.8) | 94,672 (33.4) | 53,410 (34.4) |
| Current | 46,251 (10.5) | 27,177 (9.6) | 19,074 (12.3) |
| **Heavy alcohol drinking, n (%)** | 192,908 (44.0) | 135,420 (47.8) | 57,488 (37.0) |
| **Unhealthy diet, n (%)** | 229,560 (52.4) | 144,903 (51.2) | 84,657 (54.5) |
| **Low physical activity, n (%)** | 61,620 (14.1) | 44,130 (15.6) | 17,490 (11.3) |
| **Sleep duration ≤6 or ≥9 h/d, n (%)** | 111,894 (25.5) | 67,750 (23.9) | 44,144 (28.4) |
| **BMI** | 27.2 ± 4.7 | 27.1 ± 4.6 | 27.6 ± 4.8 |
| **History of hypertension, n (%)** | 118,124 (26.9) | 69,045 (24.4) | 49,079 (31.6) |
| **History of diabetes, n (%)** | 22,529 (5.1) | 12,048 (4.3) | 10,481 (6.7) |
| **History of Hyperlipidemia, n (%)** | 56,125 (12.8) | 31,159 (11.0) | 24,966 (16.1) |
| Data are presented as mean ± standard deviation or n (%). For dichotomous variables, only the indicated category is presented. Abbreviations: BMI, body mass index; h/d, hours/day; SDOH, social determinants of health; FDBJD, first degenerative bone and joint diseases. | | | |

| **Table S22. Associations of social determinants of health with different transitions by multi-state model after multiple imputation of missing SDOH variables and covariates.** | | | | | | | | | | |
| --- | --- | --- | --- | --- | --- | --- | --- | --- | --- | --- |
| **Overall SDOH** | **Baseline to FDBJD** | | **Baseline to Death** | | **FDBJD to DBJM** | | **FDBJD to Death** | | **DBJM to Death** | |
|  | **HR (95% CI)** | ***P* value** | **HR (95% CI)** | ***P* value** | **HR (95% CI)** | ***P* value** | **HR (95% CI)** | ***P* value** | **HR (95% CI)** | ***P* value** |
| Favourable [0-5] | 1.00 Ref. |  | 1.00 Ref. |  | 1.00 Ref. |  | 1.00 Ref. |  | 1.00 Ref. |  |
| Intermediate [6-8] | **1.11 (1.09, 1.14)** | **< 0.001** | **1.18 (1.15, 1.22)** | **< 0.001** | **1.18 (1.11, 1.26)** | **< 0.001** | **1.19 (1.12, 1.28)** | **0.002** | 1.17 (0.99, 1.38) | 0.072 |
| Unfavourable [9-17] | **1.23 (1.21, 1.26)** | **< 0.001** | **1.64 (1.59, 1.69)** | **< 0.001** | **1.39 (1.31, 1.47)** | **< 0.001** | **1.67 (1.56, 1.78)** | **< 0.001** | **1.48 (1.25, 1.75)** | **< 0.001** |
| Per score point | **1.03 (1.03, 1.04)** | **< 0.001** | **1.09 (1.08, 1.09)** | **< 0.001** | **1.06 (1.05, 1.06)** | **< 0.001** | **1.09 (1.08, 1.10)** | **< 0.001** | **1.06 (1.04, 1.09)** | **< 0.001** |
| Models were adjusted for age, sex, smoking status, alcohol consumption, dietary pattern, physical activity, sleep duration, BMI, history of hypertension, diabetes, and hyperlipidemia. Notes: SDOH, social determinants of health; FDBJD, first degenerative bone and joint diseases; DBJM, degenerative bone and joint multimorbidity; BMI, body mass index; HR, hazard ratio; CI, confidence interval. | | | | | | | | | | |
